# Quality Characteristics of Semi-Dried Restructured Jerky Processed Using Super-Heated Steam

**DOI:** 10.3390/foods10040762

**Published:** 2021-04-02

**Authors:** Se-Myung Kim, Tae-Kyung Kim, Hyun-Wook Kim, Samooel Jung, Hae In Yong, Yun-Sang Choi

**Affiliations:** 1 Research Group of Food Processing, Korea Food Research Institute, Wanju 55365, Korea; 50043@kfri.re.kr (S.-M.K.); privacykin@naver.com (T.-K.K.); awsm_y@kfri.re.kr (H.I.Y.); 2 Department of Animal Science & Biotechnology, Gyeongnam National University of Science and Technology, Jinju 52725, Korea; hwkim@gntech.ac.kr; 3 Division of Animal and Dairy Science, Chungnam National University, Daejeon 34134, Korea; samooel@cnu.ac.kr

**Keywords:** semi-dried, restructured jerky, super-heated steam, shear force, water activity

## Abstract

Moisture content and water activity play important roles in extending the shelf life of dried meat products, such as jerky. However, the commonly used hot air drying process is time-consuming, costly, and adversely affects the quality of dried meat products, warranting the development of an advanced and economical drying method. This study investigated the effect of super-heated steam (SHS) drying on the quality characteristics of semi-dried restructured jerky as a measure to prevent the excessive quality deterioration of meat products during drying. The control sample was dried using hot air, and the treatment samples were dried using SHS at different temperatures (200, 250, and 300 °C) and for different durations (90, 105, and 120 min). With increasing SHS temperature and duration, the moisture content, water activity, and residual nitrite content of the jerky were reduced. The shear force values for treatments at 200 and 250 °C were lower than those for the control. With a non-significant difference in lipid oxidation compared with the control, the overall acceptability score was the highest for the treatment at 250 °C for 120 min. In conclusion, SHS (250 °C for 120 min) drying has a potential industrial value to replace the hot air drying method.

## 1. Introduction

Restructured meat products are formulated from ground meat used to process products with a consistent texture and appearance [1]. The quality of restructured meat products can be improved based on various factors, including processing conditions [2], raw materials [3], and additives [4]. Thus, restructured meat products are advantageous as they can be easily processed into uniform products.

Jerky, a nutritious and convenient popular dried meat product, is in high demand among consumers [1]. The primary concern in dried meat products is the control of moisture content and water activity, which play an important role in inhibiting microbial growth and extending shelf life [5,6]. However, hot air drying, the most commonly used industrial food-drying method [7], which incurs a high energy cost and is a time-consuming process due to the low thermal conductivity of food materials [8,9]. In addition, when making jerky by using hot air drying, problems such as off-flavor and tough texture arise [7]. Therefore, an economic and advanced drying method is needed to maintain and/or improve the quality characteristics of jerky during the drying process.

Super-heated steam (SHS) is a newly developed technology that refers to high-temperature steam at 100–400 °C and is a drying method that evaporates moisture from the inside of the product at the boiling point without diffuse resistance [10]. SHS can save energy as high as 50–85% over hot air drying by recycling the evaporated high-temperature steam [11]. Furthermore, by condensing the exhaust steam, it is possible to collect dust and toxic compounds instead of releasing them into the environment and, thus, minimize the exhaust of environmentally polluting materials [12,13]. Compared to hot-air, SHS is an oxygen free environment, thus minimizing the loss of easily oxidized nutrients such as vitamin C [14]. SHS drying can also improve the quality of dried products, including texture and rehydration behavior [4,15]. However, burn-out induced by overheating is one of the disadvantages of SHS, which limits its use as a suitable industrial drying method.

Therefore, the present study was aimed at identifying an optimal drying condition for SHS. We investigated the effects of SHS drying at different steam temperatures (200, 250, and 300 °C) for different durations (90, 105, and 120 min) on the quality characteristics of semi-dried restructured jerky. These results may provide theoretical guidance for optimizing the processing technology of SHS drying.

## 2. Materials and Methods

### 2.1. Preparation of Restructured Jerky

Fresh pork ham (musculus semimembranosus, musculus semitendinosus, and musculus biceps femoris) were purchased from a local butcher’s shop (Jeonju, Korea) at 48 h postmortem. Pork ham was removed from the visible connective tissue and ground using a meat grinder (SMC-22A, SL Machinery, Seoul, Korea) through an 8 mm plate. The semi-dried restructured jerky consisted of the following components for raw meat weight (10 kg, w/w): salt (1.2%), phosphate (0.20%), duck skin gelatin (1%), carrageenan (0.30%), ascorbic acid (0.03%), sodium nitrite (0.005%), sugar (2%), soy sauce (3%), onion powder (0.18%), garlic powder (0.18%), and black pepper powder (0.18%). The ground lean meat and salt were homogenized for 1 min in a silent cutter (Nr-963009, Hermann Scharfen & Co., Witten, Germany) and then homogenized by adding phosphate, duck skin gelatin, soy sauce, and ice. Then, the rest of the ingredients were homogenized into a batter, and the temperature of the meat batter was adjusted to 15 °C. The meat batter was stuffed into a cellulose casing (Viskase Sales Co., Chicago, IL, USA) that was 20 mm in diameter, and the length was set at 20 cm each.

### 2.2. Drying of Restructured Jerky

All samples were heated at 55 °C for 90 min in a chamber (MAXi35012 chamber, Kerres, Postfash, Germany), followed by the removal of the casing. The samples were then divided into two groups. The first group (control) was dried with hot air as follows: 55 °C for 30 min, 65 °C for 180 min, and 80 °C for 60 min in a chamber [1]. The second group was dried using a super-heated steamer (QF-5200C, Naomoto, Osaka, Japan) under ambient pressure. The evaporation rate and heater power of the super-heated steamer were kept constant at the maximum value of 9.45 kg/h and 6.65 kW, respectively. The treatments were adjusted to different steam temperatures (200, 250, and 300 °C) for different durations (60, 90, and 120 min) and at constant temperature (100 °C) for the oven. During the SHS durations, the sample was turned over after halfway through the process to ensure uniform treatment. The procedure was performed in triplicate for each sample.

### 2.3. Analysis of Moisture Content and Water Activity

The moisture content was measured using the oven drying method (at 105 °C for 24 h) [16]. The water activity of the restructured jerky sample (3 g) was determined using a water activity meter (Labmaster-aw, Novasina, Lachen, Switzerland).

### 2.4. Analysis of Processing Yield

The processing yield was determined from the weight differences of the jerky before and after drying using the following equation [1].

Processing yield (%) = [Weight after drying (g)/Weight before drying (g)] × 100(1)

### 2.5. Scanning Electron Microscopy (SEM)

The semi-dried restructured jerky was cut into two pieces (0.5 × 0.5 × 0.2 cm) for SEM analysis. Each sample was fixed with 2 mL Karnovsky’s fixative (50% glutaraldehyde, 16% formaldehyde, and 0.2 M phosphate buffer) at 4 °C for 4 h and washed with 0.05 M sodium cacodylate buffer at room temperature for 10 min, and repeated three times. Samples were fixed with 0.2% osmium tetroxide in 0.1 M sodium cacodylate buffer for 2 h at 4 °C in a dark room and washed with distilled water twice. Samples were then dehydrated using different concentrations (30, 50, 70, 80, 90, and 99.8%) of ethyl alcohol for 10 min. Each sample was dehydrated for critical drying with 99.8% ethyl alcohol for 1 h, covered with aluminum stubs, and coated with a platinum layer under vacuum (E-1010, HITACHI, Tokyo, Japan). Micrographs of the semi-dried restructured jerky with hot air and SHS drying were obtained under a scanning electron microscope (S-2380N, HITACHI, Tokyo, Japan).

### 2.6. Analysis of Shear Force

The jerky samples were cut into pieces of 3 cm, according to Kim et al. [17], and the shear force (kg) was measured using a TA-XT plus texture analyzer (Stable Micro Systems Ltd., Surrey, UK) with a test speed of 2 mm/s and a thin Warner–Bratzler blade with a V slot.

### 2.7. Analysis of Residual Nitrite Content

Residual nitrite was determined by the nitrite that remained in the final meat product without being converted into other substances [18]. Ground restructured jerky (1 g) was placed in individual 50 mL tubes, to which approximately 35 mL of distilled water was added, and heated at 80 °C in a water bath (JSSB-30T, JS Research Inc., Gongju, Korea) for 2 h. The heated solution was then cooled and filtered using Whatman No. 1 filter paper, followed by dilution of the filtrate with distilled water to a final volume of 100 mL in a 100 mL flask. To 10 mL of the sample solution, 2.5 mL of sulfanilamide reagent and 2.5 mL of N-(1-naphthyl)-ethylenediamine dihydrochloride regent was added in a 50 mL volumetric flask, diluted up to 50 mL with distilled water and allowed to stand for 15 min. The absorbance was measured at 540 nm using a microplate reader (Spectra Max Plus 384, Molecular Devices Inc., San Jose, CA, USA). The concentration of nitrite was calculated using the nitrite standard curve.

### 2.8. Determination of pH

The pH was measured with a pH meter (Mettler-Toledo GmbH., Schwerzenbach, Switzerland) calibrated with standard buffers of pH 4, 7, and 10. Samples (5 g) were homogenized at 8000 rpm with 20 mL of distilled water, and then the pH was measured.

### 2.9. Analysis of Rehydration Capacity

The rehydration capacity was measured following the method described by Kim et al. [3]. Samples were cut into 2.0 × 2.0 × 2.0 mm^3^ volume, and 100 mL of distilled water was added to a 200 mL beaker. The sample was weighed before and after 15, 30, 45, and 60 min of rehydration. The rehydration rate was calculated as follows:

Rehydration capacity (%) = [Weight of the sample after swelling (g)/Weight of the sample before swelling (g)] × 100(2)

### 2.10. Color Determination

A colorimeter (CR-410, Minolta Ltd., Tokyo, Japan) was used to measure the semi-dried restructured jerky’s surface. Illuminate C, and a 2° observer angle were set up in the equipment. The jerky was cut into pieces of 3 cm, and the surface was peeled off for the experiments. Samples were evaluated for the color of the surface at different locations. CIE L* (lightness), CIE a* (redness, ± red-green), and CIE b* (yellowness, ± yellow-blue) values were recorded.

### 2.11. Analysis of Sulfhydryl Concentration

The sulfhydryl concentration representing the protein oxidation was estimated following the method described by Berardo et al. [2]. One gram of semi-dried restructured jerky was homogenized at 8000 rpm for 30 s with 20 mL of 5% sodium dodecyl sulfate (SDS) in 0.1 M Tris(hydroxymethly)-aminomethane (TRIS) buffer (pH 8.0) followed by incubation at 80 °C in a water bath for 30 min. After cooling, the sample was centrifuged at 1200× *g* for 20 min at 4 °C and filtered through Whatman No. 1 filter paper. To measure the protein concentration, a solution containing 1 mL of the supernatant and 5 mL of 0.1 M TRIS buffer (pH 8.0) was analyzed spectrophotometrically at 280 nm using a bovine serum albumin standard curve. One milliliter of supernatant was added to 1 mL of 10 mM 5,5′-dithio-bis-2-nitrobenzoic acid (DTNB) dissolved in 4 mL of 0.1 M TRIS buffer (pH 8.0). Solutions containing 1 mL of 5% SDS in a 0.1 M TRIS buffer, with 1 mL of 10 mM DTNB dissolved in 4 mL of 0.1 M TRIS buffer, were used as the reagent blank. All solutions had a reaction time of 30 min in the dark, and the absorbance (Abs) of the sulfhydryl content was measured at 412 nm using a microplate reader (Spectra Max Plus 384). The sulfhydryl concentration was calculated as follows:

Sulfhydryl concentration (nmol/mg protein concentration) = [(Abs 412/ε)/protein concentration (nmol/mg protein)],(3)

where the molar extinction coefficient (ε) is 14,000 M^−1^ cm^−1^.

### 2.12. Analysis of Thiobarbituric Acid Reactive Substances (TBARS) Values

To analyze the lipid oxidation, TBARS values were measured according to Ohkawa et al. [19]. Five grams of the restructured jerky sample was homogenized with 15 mL of distilled water and 50 μL of 0.3% butylated hydroxytoluene (Sigma-Aldrich, St. Louis, MO, USA) in 99.8% methanol (Daejung Co., Goryeong, Korea) and transferred to a distillation flask. The homogenate (1 mL) was transferred to a test tube and mixed with 2 mL of 0.02 M thiobarbituric acid (Sigma-Aldrich) in 15% trichloroacetic acid (Junsei Chemical Co., Ltd., Tokyo, Japan) solution. The test tubes were heated at 90 °C in a water bath (JSSB-30T) for 30 min, cooled for 30 min in tap water (15 °C), and centrifuged (Combi R515) at 3100× *g* for 10 min. The absorbance of the supernatant was measured at 532 nm using a microplate reader (spectra max Plus 384) against a blank sample containing 1 mL distilled water and 2 mL thiobarbituric acid solution. The amount of malondialdehyde (MDA) was calculated using a standard curve, and the TBARS value was estimated as mg MDA per kg of sample.

### 2.13. Sensory Evaluation

The sensory characteristics of the semi-dried restructured jerky were analyzed by a panel of 33 experts who had at least 1 year of previous experience in sensory evaluation testing on this product. The samples were cut into 1 cm long sections and offered to the panels to evaluate the sensory characteristics using a 9-point hedonic scale (1 = extremely undesirable, 9 = extremely desirable), including color, flavor, texture, juiciness, and overall acceptability [20]. After each sample, the panelists were asked to cleanse their palate with warm water.

### 2.14. Statistical Analysis

All experimental data were analyzed using the SPSS statistical software program (SPSS Ver. 20.0; IBM Inc., Chicago, IL, USA). The results of the three independent replicates are presented as mean ± standard deviation (SD). The significance of differences among mean values was determined by Duncan’s multiple range tests with a confidence level of *p* < 0.05. A multifactorial analysis of variance using a general linear model procedure was applied to investigate the effects of SHS drying methods at different temperatures (200, 250, and 300 °C) and durations (90, 105, and 120 min). The significance effects of the SHS temperature, SHS duration, and SHS temperature × SHS duration was shown at confidence level of *p* < 0.001, *p* < 0.01, and *p* < 0.05, which is a smaller p value represents a more significant impact.

## 3. Results and Discussion

### 3.1. Moisture Content, Processing Yield, and Water Activity

The moisture content, processing yield, and water activity of the semi-dried restructured jerky processed with SHS are presented in Table 1. SHS temperature, SHS duration, and their interactions (SHS temperature × SHS duration) significantly affected the moisture content and processing yield of jerky, respectively (*p* < 0.001)—the moisture content and processing yield of the jerky samples processed at 300 °C were lower than those of the control (*p* < 0.05). These results indicated that high SHS temperature rapidly evaporates the moisture; hence, reducing the processing yield of jerky.

The drying process involves three different stages: (i) the initial heating period, (ii) the constant rate period, and (iii) the falling rate period [21]. During the (ii) constant rate period, free water evaporates from the product, reducing the moisture content and water activity of the product [22]. In hot air drying, water diffuses from the product to the air through the boundary layer of water surrounding the product [21], which encounters the diffusive resistance in water movement through the boundary layer. However, in SHS drying, water only moves by mass flow, and thus does not encounter this resistance [13,21]; therefore, the drying rate of SHS is faster than that of hot air drying. Moreover, in the (ii) constant rate period, there exists an inversion temperature, above which the evaporation rate is higher in SHS drying than in hot air drying [23]. Taken together, it can be inferred that SHS temperature above the inversion temperature imposes a greater driving force of temperature, and hence a larger heat flux, consequently leading to a higher evaporation rate of the moisture [24]. Similar results were reported by Speckhahn et al. [7], who showed that the moisture content of minced meat decreased faster through SHS drying compared to hot air drying at the same temperature. It has also been reported that SHS temperature and processing time significantly affected the moisture of chicken breast fillets [25]. Drying is important for extending the shelf life of intermediate moisture foods (IMFs) such as semi-dried jerky by reducing free water and inhibiting microbial growth [26]. In the present study, SHS at 200 °C revealed a higher moisture content (50%) of jerky, which was above the normal moisture content of the IMF (20–50%; [27]); suggesting that 200 °C might not be the optimum temperature for drying semi-dried restructured jerky.

Similarly, the water activity of the jerky decreased significantly with increasing SHS temperature and duration (*p* < 0.001), and was lowest with SHS at 300 °C for 120 min (Table 1), which could be attributed to the decrease in free water during the constant rate [22], or the reduced moisture content resulting from the rapid evaporation of water at high SHS temperature [28]. Water activity is one of the most important factors associated with the classification criteria for dried meat products [29], and a water activity of 0.60–0.90 in semi-dried food is considered relatively safe from microorganisms [30]. However, we detected a water activity of 0.919–0.921, indicating that SHS at 200 °C cannot be applied to dry semi-dried jerky. Collectively, the results of the present and the previous studies indicated that processing efficiency could be improved by reducing the dehydration time of the sample [15].

### 3.2. SEM

**Figure 1:**
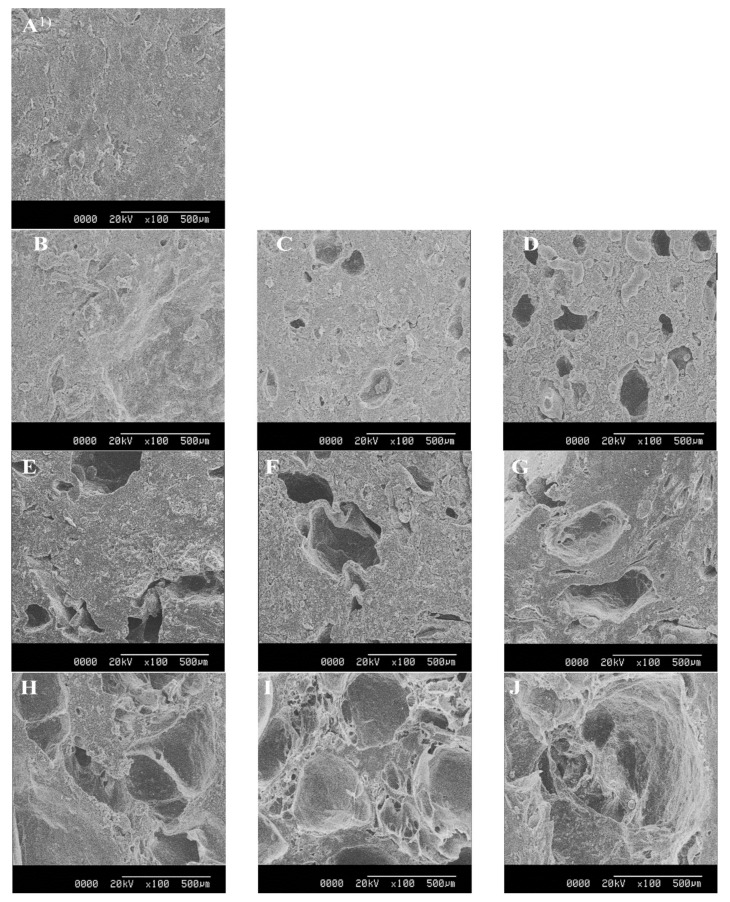
Scanning electron microscopy of semi-dried restructured jerky processed with super-heated steam (SHS) (**A**), hot air drying (control); (**B**–**D**) super-heated steam drying at 200 °C for 90, 105, and 120 min; (**E**–**G**) super-heated steam drying at 250 °C for 90, 105, and 120 min; (**H**–**J**) super-heated steam drying at 300 °C for 90, 105, and 120 min.

SEM images of semi-dried restructured jerky prepared by SHS are shown in Figure 1. In the falling rate period of the drying process, there was no difference in the cross-sections between the control and the samples dried with SHS at 200 °C for 90 min (Figure 1A,B). In semi-dried restructured jerky with SHS, it was confirmed that, with increasing SHS temperature, more cracks occurred in the cross-sections, and the size of the cracks gradually increased with increasing SHS duration, which could be due to the rapid moisture loss with the increased internal temperature of the samples at the early stages of drying [24]. As previously discussed, at the falling rate period in the drying process, the temperature of the product will rise to that of the SHS [31]. The drying rate is greater for SHS than hot air drying because the product’s higher temperature allows for higher moisture diffusion [21]. Due to this phenomenon, the products dried in SHS are more porous than those of hot air drying [21,32]. Our results were consistent with those of Jamradloedluk et al. [33], who reported a higher number of pores in durian chips dried with SHS than those dried with hot air. In addition, similar effects of higher SHS temperature have been reported in tortilla chips [34].

### 3.3. Shear Force

As shown in Table 1, the value of the sheer force of the jerky samples was significantly affected by SHS temperature, SHS duration, and their interactions (*p* < 0.001). It was reduced by most of the SHS temperatures and durations except for SHS at 300 °C for 105 min (the reduction was not significant) and 120 min (*p* < 0.05). In addition, the control showed a higher shear force value than all SHS treatments except for SHS at 300 °C for 105 min (the reduction was not significant) and 120 min (*p* < 0.05). In hot air drying, the case hardening creates a boundary layer by preventing the inner moisture from reaching the surface, which generates severe shrinkage and a tough texture of the product [35]. However, in SHS drying, the shear force value of the dried product is lowered by preventing the case hardening [31]. The increase in shear force could be related to decreased moisture content with increased SHS temperature and duration (Table 1). Moreover, once the surface of the product is dried, the moisture diffusion inside becomes faster due to the high SHS temperature, leading to an expansion of the volume of the product and a rapid decrease in the moisture content [32,36]. A recent study reporting the drying of semi-dried whole wheat noodles with SHS has demonstrated increased shear force with increasing SHS temperature [37]. Similar effects of SHS on the shear force have been reported in chicken steak [4] and lamb patties [38].

### 3.4. Residual Nitrite, pH, and Color

As shown in Table 2, both SHS temperature and SHS duration significantly affected residual nitrite content (*p* < 0.001 and 0.05, respectively); the highest residual nitrite content was found in the control (12.18 ppm), and the lowest in treatments dried with SHS at 300 °C (1.78–2.54 ppm; *p* < 0.05). Considering the same SHS temperature, no significant (*p* > 0.05) difference was observed in residual nitrite content at different SHS durations. These results suggested that the decomposition of nitrite was promoted by the SHS temperature but not by the SHS duration. The decrease in the residual nitrite levels with processing such as heating, curing, and packaging has also been reported in earlier studies [39,40]. The heating process, in particular, is observed to result in a loss of 20–80% of the initial nitrite [41,42]. Here, we observed significantly lower residual nitrite content in all jerky samples when compared to the control samples (*p* < 0.05), which were consistent with the findings of Li et al. [43], who showed that heating depleted the residual nitrite content by about 65% of the initially added nitrite. Moreover, the reduced residual nitrite content of semi-dried restructured jerky processed with SHS could be advantageous in terms of reduced nitrosamine formation, as the residual nitrite can react with amines and amino acids to produce nitrosamines, which are known to be teratogenic, mutagenic, and carcinogenic [44]. However, nitrosamines can be formed when nitrites and amines are heated above 130 °C in meat products [41], which are conditions that jerky processed with SHS may experience.

Furthermore, both SHS temperature and SHS duration significantly affected the pH of semi-dried restructured jerky (*p* < 0.001; Table 3); the pH of the jerky samples decreased with increasing SHS temperature and duration (*p* < 0.05). These decreases might be due to protein unfolding through denaturation and the binding or release of protons from protein [45]. Additionally, the loss of positive charges on the protein molecule by the Maillard reaction during the drying process could also be a contributing factor [46]. Furthermore, our findings are supported by those of Mahdy and Yang [47], who reported a significant decrease in the pH value of milk powder with increased SHS temperature and time during the drying process.

The L*, a*, and b* values of the surface of the semi-dried restructured jerky processed with SHS are shown in Table 2. SHS temperature and SHS duration significantly affected the L*and b* values of jerky, respectively (*p* < 0.001). All SHS treatments revealed a higher L* value than the control (*p* < 0.05). The L* and b* values of the jerky samples decreased with increasing SHS temperature and duration (*p* < 0.05). However, except for SHS at 300 °C, there was no significant difference in a* values between the SHS-treated and control samples (*p* > 0.05). The decrease in L* values could be due to the significant structural changes in jerky proteins during SHS drying at a higher temperature [48]. These trends of color change could be due to the combined effects of the oxygen-free environment in SHS treatment and the rapid temperature rise inside the meat, which initiates the Maillard browning reactions [24]. Furthermore, it could also be due to the reaction between amine groups of muscle proteins and available reducing sugars in connective tissues during the drying process of meat products [10,48]. In congruence, Speckhahn et al. [7] showed that increasing temperature and duration of SHS decreased L* and b* values of dried beef slices. Additionally, Suleman et al. [38] reported similar results of decreased L*, a*, and b* values with increasing SHS temperature and frying time of Gulab Jamun balls.

### 3.5. Sulfhydryl Concentration and TBARS

The sulfhydryl concentration and TBARS values of semi-dried restructured jerky processed with SHS are shown in Table 3. With an increased SHS temperature and duration, the sulfhydryl concentration of the jerky samples was reduced (*p* < 0.001), except for the jerky processed with SHS at 200 °C (*p* < 0.05 vs. control). In general, when meat products are cooked at high temperatures, protein crosslinking occurs through the formation of disulfide bridges, resulting in a decreased sulfhydryl concentration [49]. Therefore, the decreased sulfhydryl concentration of semi-dried restructured jerky could be associated with the high SHS temperature. Additionally, an increased sulfhydryl concentration due to excessive heat treatment can increase the toughness of meat products [50]; however, our results were not influenced by this mechanism, as the SHS at 250 °C treatment group, which showed a relatively higher sulfhydryl concentration than the control, had lower shear force (Table 1).

It was observed that none of the SHS treatments, except for SHS at 200 °C for 90 min, had a significant effect on the TBARS of semi-dried restructured jerky (*p* > 0.05; Table 3), indicating no influence of SHS on lipid oxidation. Lipid oxidation, a major cause of deterioration in the quality of food and food products, generally oxidizes unsaturated fatty acids, producing free radicals at high temperatures in the presence of oxygen [51,52]. With the temperature being one of the major factors influencing lipid oxidation, SHS, with a higher temperature than the hot air drying method, could promote lipid oxidation; however, the non-significant influence of SHS on lipid oxidation could be due to the absence of oxygen content in the SHS used for the drying process. These findings were consistent with the findings of Huang et al. [53], who reported that Zousoon fried with SHS had higher lipid stability than the pan-frying method.

### 3.6. Rehydration Rate

**Figure 2:**
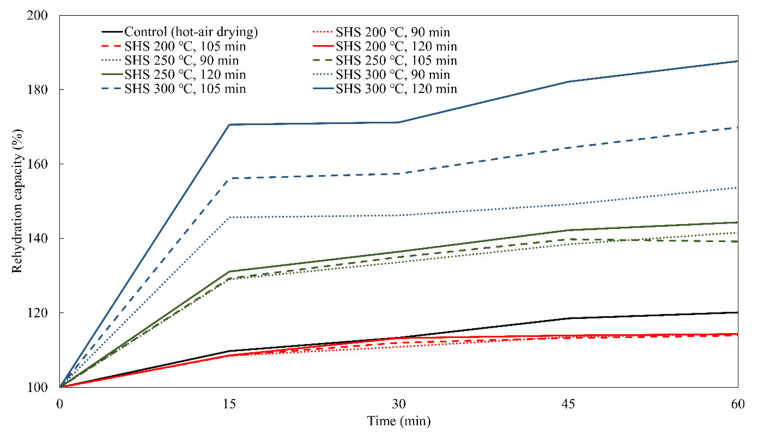
Rehydration capacity of semi-dried restructured jerky processed with super-heated steam (SHS).

The rehydration rates of the semi-dried restructured jerky processed with SHS are shown in Figure 2. The results revealed that the rate of rehydration increased with increasing SHS temperature and duration. The rehydration rates of the jerky processed with SHS at 250 and 300 °C was higher than those of the control, which might improve the toughness of the jerky. Nathakaranakule et al. [10] reported that the SHS temperature increased the rehydration rate in cube-type chicken meat processing. Jamradloedluk et al. [33] also demonstrated that durian chips dried with SHS had a higher rehydration rate than those dried with hot air. The increased rehydration rate could be explained by the porous structure of the SHS samples (Figure 1), consequently absorbing more water.

### 3.7. Sensory Characteristics

The sensory characteristics of the semi-dried restructured jerky processed with SHS are shown in Table 4. SHS temperature significantly affected the color (*p* < 0.01), flavor, texture, juiciness, and overall acceptability (*p* < 0.001). There were no significant differences between the control and SHS treatments for color and flavor (*p* > 0.05). The development of the undesirable flavor of meat products is affected by lipid oxidation [54]. In the present study, since the TBARS of the jerky showed no significant difference (Table 3), the flavor of the restructured jerky showed no significant difference among the treatments. The texture of the jerky processed with SHS at 200 °C for all durations and 250 °C for 90 min were significantly different from the control (*p* < 0.05). These observed changes in texture values could be related to increased shear force with increased SHS temperature and duration (Table 1). Jerky processed with SHS at 250 °C for 120 min had the highest overall acceptability scores (*p* < 0.05). These results were in accordance with those of Choi et al. [4], who reported that SHS-cooked meat has better tenderness, juiciness, and overall acceptability scores than that cooked by boiling, steaming, grilling, or microwaving. Furthermore, the juiciness scores of jerky processed with SHS were decreased with increasing SHS temperature, which was consistent with the moisture content results (Table 1). According to Suleman et al. [51], water loss during drying decreases the juiciness of the final meat product. Additionally, Isleroglu et al. [55] reported that SHS temperature ranging from 150 to 250 °C improved the texture of lamb patties. These studies indicated that too high temperatures, such as SHS at 300 °C, could degrade the texture and reduce the juiciness of semi-dried restructured jerky; however, SHS at 250 °C for 120 min demonstrated excellent sensory characteristics of semi-dried restructured jerky.

## 4. Conclusions

In summary, the findings of the present study demonstrated the suitability of SHS as a potential and advanced method for drying in the restructured-jerky manufacturing process. Particularly, SHS at 250 °C for 120 min were identified as the optimum conditions, maintaining most of the quality attributes, including water activity, shear force, residual nitrite content, and sensory properties. Additionally, SHS technology has technical and environmental advantages, as it could save energy by recycling the high-temperature steam and minimize polluting emissions. Collectively, SHS could be an eco-friendly, advanced drying method to replace the hot air drying method employed in the drying process of restructured jerky without significant quality changes, and therefore have potential industrial value.

## Figures and Tables

**Table 1:** Moisture content, processing yield, water activity, and sheer force of semi-dried restructured jerky processed with super-heated steam (SHS).

Treatments		Moisture Content (%)	Processing Yield (%)	Water Activity	Shear Force (kg)
Control	-	40.19 ± 0.49 ^e^	59.51 ± 0.05 ^e^	0.867 ± 0.001 ^d^	15.11 ± 0.38 ^b^
SHS 200 °C	90 min	53.52 ± 0.15 ^a^	76.44 ± 1.04 ^a^	0.921 ± 0.005 ^a^	7.92 ± 0.11 ^f^
	105 min	53.27 ± 0.30 ^a^	75.40 ± 0.84 ^a^	0.919 ± 0.003 ^a^	8.19 ± 0.38 ^f^
	120 min	51.74 ± 0.55 ^b^	72.09 ± 1.20 ^b^	0.919 ± 0.003 ^a^	8.26 ± 0.28 ^f^
SHS 250 °C	90 min	46.33 ± 1.26 ^c^	66.01 ± 0.28 ^c^	0.899 ± 0.003 ^b^	8.47 ± 0.20 ^e^
	105 min	45.09 ± 1.29 ^c^	63.05 ± 0.85 ^d^	0.893 ± 0.002 ^c^	9.28 ± 0.33 ^d^
	120 min	41.93 ± 1.07 ^d^	62.29 ± 0.84 ^d^	0.890 ± 0.002 ^c^	10.19 ± 0.48 ^d^
SHS 300 °C	90 min	37.72 ± 0.25 ^f^	58.54 ± 0.58 ^e^	0.867 ± 0.002 ^d^	12.27 ± 0.19 ^c^
	105 min	31.44 ± 0.14 ^g^	52.83 ± 0.89 ^f^	0.813 ± 0.003 ^e^	14.46 ± 0.69 ^b^
	120 min	24.51 ± 0.93 ^h^	47.26 ± 0.72 ^g^	0.776 ± 0.001 ^f^	18.16 ± 0.41 ^a^
*p*-Value					
SHS temperature		***	***	***	***
SHS duration		***	***	***	***
SHS temperature × SHS duration		***	***	***	***

Data are shown as the mean ± SD. The different letters in the same line represent significant differences in the results of the Duncan test (*p* < 0.05). *** *p* < 0.001.

**Table 2:** Residual nitrite, pH, and color of semi-dried restructured jerky processed with super-heated steam (SHS).

Treatments		Residual Nitrite (ppm)	pH	CIE L*	CIE a*	CIE b*
Control	-	12.18 ± 0.08 ^a^	6.14 ± 0.01 ^fg^	36.81 ± 0.66 ^h^	14.00 ± 0.25 ^b^	15.50 ± 0.36 ^f^
SHS 200 °C	90 min	4.68 ± 0.78 ^b^	6.24 ± 0.03 ^a^	45.52 ± 0.04 ^a^	14.50 ± 0.35 ^a^	23.85 ± 0.10 ^a^
	105 min	4.46 ± 0.41 ^b^	6.23 ± 0.02 ^a^	44.76 ± 0.48 ^b^	14.22 ± 0.13 ^ab^	23.69 ± 0.66 ^a^
	120 min	3.88 ± 0.08 ^b^	6.18 ± 0.01 ^cd^	44.60 ± 0.35 ^b^	14.21 ± 0.39 ^ab^	23.03 ± 0.10 ^a^
SHS 250 °C	90 min	4.19 ± 0.77 ^b^	6.20 ± 0.01 ^b^	43.28 ± 0.73 ^c^	14.42 ± 0.29 ^ab^	21.76 ± 0.72 ^b^
	105 min	4.10 ± 0.54 ^b^	6.19 ± 0.01 ^bc^	42.98 ± 0.49 ^cd^	14.40 ± 0.18 ^ab^	20.69 ± 0.74 ^c^
	120 min	3.79 ± 0.54 ^b^	6.16 ± 0.01 ^de^	42.40 ± 0.46 ^de^	13.96 ± 0.31 ^b^	19.39 ± 0.56 ^d^
SHS 300 °C	90 min	2.54 ± 0.08 ^c^	6.15 ± 0.01 ^ef^	41.90 ± 0.21 ^e^	12.49 ± 0.13 ^c^	18.63 ± 0.40 ^d^
	105 min	2.45 ± 0.08 ^c^	6.12 ± 0.01 ^gh^	40.17 ± 0.32 ^f^	11.73 ± 0.20 ^d^	16.83 ± 0.52 ^e^
	120 min	1.78 ± 0.39 ^c^	6.11 ± 0.01 ^h^	38.50 ± 0.07 ^g^	11.76 ± 0.21 ^d^	15.58 ± 0.57 ^f^
*p*-Value						
SHS temperature		***	***	***	**	***
SHS duration		*	***	***	***	***
SHS temperature × SHS duration		NS	***	***	NS	*

Data are shown as the mean ± SD. The different letters in the same line represent significant differences in the results of the Duncan test (*p* < 0.05). * *p* < 0.05, ** *p* < 0.01, *** *p* < 0.001; NS, no significance.

**Table 3:** Protein oxidation and lipid oxidation values of semi-dried restructured jerky processed with super-heated steam (SHS).

Treatments		Sulfhydryl Concentration (nmol/mg)	TBARS (mg MDA/kg)
Control	-	48.27 ± 0.23 ^d^	0.45 ± 0.01 ^ab^
SHS 200 °C	90 min	65.66 ± 0.45 ^a^	0.31 ± 0.08 ^c^
	105 min	59.68 ± 0.27 ^b^	0.36 ± 0.12 ^bc^
	120 min	51.58 ± 0.04 ^c^	0.41 ± 0.02 ^ab^
SHS 250 °C	90 min	37.53 ± 0.11 ^e^	0.43 ± 0.04 ^ab^
	105 min	34.86 ± 0.60 ^f^	0.45 ± 0.05 ^ab^
	120 min	20.18 ± 0.03 ^i^	0.48 ± 0.03 ^a^
SHS 300 °C	90 min	26.79 ± 0.38 ^g^	0.48 ± 0.02 ^a^
	105 min	23.43 ± 0.46 ^h^	0.48 ± 0.07 ^a^
	120 min	18.54 ± 0.78 ^j^	0.50 ± 0.01 ^a^
*p*-Value			
SHS temperature		***	**
SHS duration		***	NS
SHS temperature × SHS duration		***	NS

Data are shown as the mean ± SD. The different letters in the same line represent significant differences in the results of the Duncan test (*p* < 0.05). ** *p* < 0.01, *** *p* < 0.001; NS, no significance.

**Table 4:** Sensory evaluation in semi-dried restructured jerky processed with super-heated steam (SHS).

Treatments		Color	Flavor	Texture	Juiciness	Overall Acceptability
Control	-	6.18 ± 1.36 ^a^	6.64 ± 1.39 ^a^	4.30 ± 0.64 ^e^	4.79 ± 0.86 ^b^	5.88 ± 1.20 ^bc^
SHS 200 °C	90 min	4.94 ± 1.94 ^b^	5.76 ± 1.12 ^abc^	6.94 ± 0.70 ^ab^	5.61 ± 1.00 ^a^	5.45 ± 0.83 ^c^
	105 min	5.03 ± 1.96 ^b^	6.00 ± 1.35 ^ab^	7.24 ± 0.75 ^a^	5.88 ± 1.19 ^a^	5.64 ± 1.14 ^bc^
	120 min	4.94 ± 1.84 ^b^	6.00 ± 1.03 ^ab^	6.70 ± 0.81 ^b^	5.61 ± 0.70 ^a^	5.55 ± 0.94 ^bc^
SHS 250 °C	90 min	5.79 ± 1.82 ^ab^	6.36 ± 1.45 ^a^	5.27 ± 0.63 ^c^	4.64 ± 0.55 ^bc^	5.79 ± 0.65 ^bc^
	105 min	6.06 ± 1.58 ^ab^	5.97 ± 1.67 ^ab^	4.61 ± 0.61 ^de^	4.21 ± 0.65 ^c^	6.18 ± 1.01 ^b^
	120 min	6.27 ± 2.13 ^a^	6.18 ± 1.01 ^a^	4.79 ± 1.14 ^d^	4.27 ± 0.84 ^c^	7.03 ± 1.49 ^a^
SHS 300 °C	90 min	5.88 ± 2.12 ^ab^	5.21 ± 1.41 ^bc^	4.27 ± 0.67 ^e^	3.36 ± 0.93 ^de^	5.30 ± 1.26 ^c^
	105 min	5.21 ± 2.33 ^ab^	5.06 ± 2.51 ^c^	3.06 ± 1.25 ^f^	3.06 ± 0.61 ^e^	4.70 ± 1.74 ^d^
	120 min	5.30 ± 2.57 ^ab^	5.24 ± 2.45 ^bc^	3.18 ± 1.42 ^f^	3.70 ± 0.88 ^d^	3.97 ± 1.70 ^e^
*p*-Value						
SHS temperature		**	***	***	***	***
SHS duration		NS	NS	***	**	NS
SHS temperature × SHS duration		NS	NS	***	***	***

Data are shown as the mean ± SD. The different letters in the same line represent significant differences in the results of the Duncan test (*p* < 0.05). ** *p* < 0.01, *** *p* < 0.001; NS, no significance.

## Data Availability

The data are contained within the article. The data presented in this study are available in the present article.
